# The Administration of Chloroform

**Published:** 1903-12-12

**Authors:** 


### THE ADMINISTRATION OF CHLOROFORM.

Dr. Augustus Waller,[1] in a lecture delivered in the Physiological Laboratory of the University of London called attention to the high and increasing mortality due to the administration of anæsthetics recorded by the Registrar-General. He urged the value of scientific observations as an aid to the anæsthetist in choosing the best method of practising his art, while at the same time in no way belittling the value of clinical experience. In experiments made on animals, cats and dogs chiefly, he had found that a 2 per cent. dilution of chloroform in air was a satisfactory anæsthetic. This concentration was sufficient to induce anæsthesia in any of the animals observed, from frogs and mice to human beings, and complete anæsthesia could be produced in about seven minutes and the animal kept fully anæsthetised for twelve hours or more without any disastrous occurrence. The safe anæsthetic atmosphere for inducing and maintaining anæsthesia was one containing 1 to 2 per cent. of chloroform vapour, and this could be attained in many ways either by a piece of lint or towel held at a short distance from the patient's face and sprinkled from time to time with chloroform, or by a more complicated apparatus which would deliver so many litres of air and chloroform vapour, in the required proportion at each stroke of a piston. If a higher percentage of chloroform, say 5 per cent., were used, the animal was rapidly rendered unconscious, and died from respiratory failure in about three minutes. If a very high percentage of chloroform vapour, above 10 per cent. for instance, was present in the inspired air death took place almost instantaneously from cardiac syncope. This latter degree of concentration was found present beneath the inhaler—mask or towel—when it was held firmly over the patient's or animal's mouth. Two or three inspirations of this highly concentrated anæsthetic were sufficient to poison the heart muscle. Dr. Waller summed up his conclusions thus:—"The evidence in point shows that it is necessary, and within reasonable limits of error practicable, to secure dosage in the administration of chloroform, and I should like to at once characterise and earmark that argument by the statement that successful chloroform anæsthesia requires the regular respiration of air in which the chloroform vapour is maintained between the limits of 1 and 2 per cent."

[1] Lancet, Nov. 28, 1903.

